# Deciphering the ChitoCode: fungal chitins and chitosans as functional biopolymers

**DOI:** 10.1186/s40694-021-00127-2

**Published:** 2021-12-10

**Authors:** Stefan Cord-Landwehr, Bruno M. Moerschbacher

**Affiliations:** Institute for Biology and Biotechnology of Plants, University of Münster, Schlossplatz 8, 48143 Münster, Germany

## Abstract

Chitins and chitosans are among the most widespread and versatile functional biopolymers, with interesting biological activities and superior material properties. While chitins are evolutionary ancient and present in many eukaryotes except for higher plants and mammals, the natural distribution of chitosans, i.e. extensively deacetylated derivatives of chitin, is more limited. Unequivocal evidence for its presence is only available for fungi where chitosans are produced from chitin by the action of chitin deacetylases. However, neither the structural details such as fraction and pattern of acetylation nor the physiological roles of natural chitosans are known at present. We hypothesise that the chitin deacetylases are generating chitins and chitosans with specific acetylation patterns and that these provide information for the interaction with specific chitin- and chitosan-binding proteins. These may be structural proteins involved in the assembly of the complex chitin- and chitosan-containing matrices such as fungal cell walls and insect cuticles, chitin- and chitosan-modifying and -degrading enzymes such as chitin deacetylases, chitinases, and chitosanases, but also chitin- and chitosan-recognising receptors of the innate immune systems of plants, animals, and humans. The acetylation pattern, thus, may constitute a kind of ‘ChitoCode’, and we are convinced that new in silico, in vitro, and in situ analytical tools as well as new synthetic methods of enzyme biotechnology and organic synthesis are currently offering an unprecedented opportunity to decipher this code. We anticipate a deeper understanding of the biology of chitin- and chitosan-containing matrices, including their synthesis, assembly, mineralisation, degradation, and perception. This in turn will improve chitin and chitosan biotechnology and the development of reliable chitin- and chitosan-based products and applications, e.g. in medicine and agriculture, food and feed sciences, as well as cosmetics and material sciences.

## Chitosans as a versatile family of functional biopolymers

Chitins and, in particular, chitosans, their partially deacetylated derivatives, are among the most versatile functional biopolymers. In nature, they are integral parts of complex matrices, such as arthropod cuticles or fungal cell walls, which can be highly flexible or extremely durable, even translucent or bioactive, making them outstanding biomaterials. They have gel-, film-, and fibre-forming properties; they can be nano-formulated into nanoparticles, nanocapsules, or nanofibers; they have metal ion- and protein-binding capacities; they have antibacterial and antifungal activities; they can promote plant growth and development; they can induce plant disease resistance and abiotic stress tolerance; they have blood-coagulating and wound healing properties, etc. In different countries, they are already being used for waste and drinking water purification or the clearing of beverages such as juices, beer, and wine; as a cosmetics and health care ingredient; as a probiotic animal feed additive or human food supplement; as a plant strengthener or biopesticide; and as haemostatic or wound-healing dressing in veterinary or human medicine. Potential future uses in different stages of development are as pharmaceutical drug, gene, or vaccine delivery vehicle and diverse other medical applications, e.g. in cancer treatment. Other promising uses would be possible e.g. in the paper and textile industries or as a bioplastic, but these would require larger scale production facilities than currently available. And: chitosans are environment-friendly and consumer-safe, non-toxic and non-allergenic, biodegradable and biocompatible in many systems [1].

Of course, not all chitosans possess all of these properties and bioactivities. The functional diversity is based on an equally broad structural diversity. Chitosans are linear co-polymers of glucosamine (GlcN) and *N*-acetylglucosamine (GlcNAc) residues, differing in (i) their degree of polymerisation (DP), i.e. the number of monomeric units in the molecule, (ii) their fraction of acetylation (*F*_A_), i.e. the relative abundance of GlcNAc residues, and (iii) their pattern of acetylation (PA), i.e. the sequence of GlcN and GlcNAc residues within the chain. All three parameters are known to deeply influence the physico-chemical properties and biological activities of the chitosans [2, 3]. In fact, the solubility in slightly acidic media (below ca. pH 6) is generally accepted as the defining criterion for the distinction between acid-insoluble chitins and acid-soluble chitosans. Importantly, chitosan samples are always mixtures of molecules more or less strongly differing in these parameters, so that their dispersities (*Đ*) will also significantly influence their functionalities [4].

‘First generation’ commercial chitosans were poorly defined mixtures of chitosans with often large batch-to-batch variability, but research of the past decades enabled the production of ‘second generation’ chitosans which are well-defined in terms of DP and *F*_A_, and have known random PA [5–8]. And at lab scale, even better defined, less disperse chitosans with non-random PAs are beginning to be reported, e.g. based on biotechnological production or modification processes, raising expectations for ‘third generation’ chitosans with further improved functionalities [2, 3, 9, 10].

## Why studying fungal chitosans?

The market availability of second generation chitosans with well-defined properties and functionalities and low batch-to-batch variability has led to a surge in market interest so that for the first time, demands are exceeding supplies [11]. However, chitosan production from crustacean chitin is limited by the availability of shell wastes [12, 13]. Fungal cell walls are an interesting additional source of chitin, available in more constant quality with no seasonal variation from almost sterile waste streams of the food and biotechnology industries and, at least potentially, in a more sustainable way [14]. An additional advantage of fungal chitin over shellfish chitin is its non-animal origin which is preferred for e.g. cosmetic applications or by customers who follow a vegan lifestyle.

The main drawback of fungi as a source of chitin is the fact that in fungal cell walls, chitin is covalently linked to glucans, making extraction more demanding and the product, at least potentially, less pure [15]. Clearly, more research into the biosynthesis, cross-linking, and degradation of chitin in fungal cell walls is required as a basis to develop yield- and cost-efficient protocols for its extraction [16, 17]. And of course, a fundamental understanding of chitin metabolism and its physiological role in the growth and differentiation of fungi is an exciting scientific goal in itself.

But fungi cannot only serve as a source of chitin for chemical conversion into chitosan. Fungi are also the only confirmed source of natural chitosans [18, 19]. This biogenic chitosan is believed to originate from chitin by the action of chitin deacetylases acting in concert with chitin synthases [20]. And it is highly likely that enzymatic deacetylation yields chitosans with different, specific patterns of acetylation, while chemically produced chitosans are invariably characterised by random acetylation patterns. As the distribution of acetyl groups along the linear chitosan chain has recently been proven to critically influence the physico-chemical properties and biological activities of chitosan oligomers and polymers [9, 10, 21–23], the study of natural chitosans and their original in muro pattern of acetylation becomes highly promising, potentially opening up completely new possibilities for chitosan-based applications [2, 3].

And of course again, aiming at a fundamental understanding of fungal chitosans is an exciting and promising research area in itself. Why do some fungi under some conditions convert some of their chitin into chitosans? And how do they do it? Why do chitin deacetylases typically occur in multigene families [24, 25]? What is the DP, *F*_A_, and PA of these natural chitosans? What are their physico-chemical properties and how do they contribute to the properties of the fungal cell walls? What is the physiological role of these chitosans in growth or morphogenesis of the fungi, or in the interaction with e.g. host organisms? Plenty of questions which await answers.

## Three advances of research in the last decade

### Bioanalytics and bioinformatics offer new tools to study and model structures and functions of chitosans

The progress from first to second generation chitosans was largely driven by the improvement of analytical tools for their structural characterisation: HPSEC-RI-MALLS for DP and *Đ*_DP_ determination, ^1^H- and ^13^C-NMR for *F*_A_ and PA analysis [5, 26–28]. Currently, new methods are being developed, most prominently based on mass spectrometry (MS), first for chitosan oligomers, and then, in the form of enzymatic-mass spectrometric fingerprinting (EMS-FP), also for chitosan polymers [4, 29, 30]. MS allows the accurate determination of DP and *F*_A_ of chitosan oligomers up to a DP of ca. 15, and of PA up to DP 6, for the entire range of *F*_A_-values [29]. Also, MS techniques automatically give insight into the dispersities in both DP (*Đ*_DP_) and *F*_A_ (*Đ*_FA_), so that mixtures of chitosan oligomers can be comprehensively described, as long as they are not too complex and the DP is not too high [31]. EMS-FP, i.e. the MS analysis of chitosan oligomer mixtures obtained upon enzymatic hydrolysis of a chitosan polymer using enzymes with known subsite preferences, allows a more accurate determination of *F*_A_ and a more detailed analysis of PA than can be achieved using NMR, and this even with sub-microgram quantities of material compared to multi-milligram amounts required for NMR [10, 30, 32]. Capillary electrophoresis (CE) emerges as a potential tool to analyse *Đ*_FA_ of chitosan polymer samples, a parameter that was hitherto not accessible to any analytical technique [33, 34]. Solid state NMR allows non-destructive insights into complex biological matrices into which chitin is intrinsically embedded, such as fungal cell walls or insect cuticles [35–40]. Unexpectedly, these studies revealed a significant role of α-1,3-glucans and, at least in *Aspergillus fumigatus*, of galactomannans in the rigid chitin/ß-1,3-glucan complex of the fungal cell wall. Interestingly, depleting the cell wall of galactomannans led to a five-fold increase in chitin content [41, 42], an observation of potentially significant impact for the biotechnological production of fungal chitin and chitosan, given that the chitin content in fungal cell walls rarely exceeds 15%.

These improvements in methods of analytical biochemistry are paralleled and supported by tremendous advances in bioinformatic tools. Among them, one of the most fascinating is the ab initio modelling of protein structures using the program AlphaFold which allows the accurate prediction of the 3D structures with atomic accuracy even of proteins for which no crystal structures exist, as a basis for docking and molecular dynamics studies for e.g. chitin- or chitosan-protein interactions [43]. These are relevant for our understanding of the biosynthesis and assembly of chitin/chitosan-containing biological matrices as well as their enzymatic modification and biodegradation and, eventually, the interaction of oligomeric breakdown products with targets and receptors e.g. of the immune systems of plants and animals [44–48].

### Solid state synthesis and recombinant enzymes offer access to fully defined chitosan oligomers

This progress in analytical tools is currently driving the development of even better characterised third generation chitosans, either biotechnologically using enzymes or chemically using automated solid-state synthesis.

The biotechnological approach makes use of the increasing number of well-characterised, regio-selective chitin deacetylases and sequence-dependent chitosan hydrolases [49–55]. The latter can yield mixtures of chitosan oligomers with partially defined acetylation patterns, as the subsite preferences of the hydrolases determine the residues at and near the reducing and non-reducing ends of the oligomeric products. The former, acting in forward or reverse mode, can yield fully defined, partially acetylated chitosan oligomers [22]. Interestingly, these enzymes can also convert high-*F*_A_ chitosan polymers into low-*F*_A_ chitosans or, in reverse mode, polyglucosamines into low-*F*_A_-, then also high-*F*_A_ chitosan polymers with, depending on the enzyme used, different PAs [9, 10].

By sequentially adding monosaccharide building blocks to a solid support, automated glycan synthesis allows the production of practically any chitosan oligomer with fully defined sequence, limited only by solubility issues particularly of high-DP, high-*F*_A_ oligomers [56, 57].

Unlike the enzymatic approach which is limited in the oligomers that can be targeted by the sequence preferences of the (still few) available enzymes, chemical synthesis can yield any oligomer. On the other hand, solid-state synthesis only yields low amounts of products in the mg-range, while enzymatic production processes can be scaled up, potentially to yield kg-amounts.

### Physicochemical properties and biological activities of chitosans are influenced by the pattern of acetylation

Theoretical considerations had long predicted that PA should play a crucial role in determining both physico-chemical properties and biological activities of chitosans, and first experimental evidence starts accumulating to verify this hypothesis.

The physico-chemical properties of chitosan polymers have been predicted to be influenced by PA based on comparison with other natural biopolymers or synthetic copolymers in which the pattern of substitution or the monomer sequence is known to influence e.g. solubility, phase behaviour, solution rheology, gelling, crystallinity, or conformation. Studies using chitosan polymers prepared using recombinant chitin deacetylases and, therefore, differing in their PA now showed that PA indeed influences the rheological properties of chitosans, including their suitability for nano-formulation [9, 10].

The influence of PA on biological activities of chitosans has been predicted based on the assumption that the sequence of more hydrophobic GlcNAc and positively charged GlcN residues will determine their interaction with hydrophobic and negatively charged patches on the surface of proteins [58]. These might be chitinases or chitosanases processing the chitosan polymers applied to a target tissue, yielding specific oligomeric products. And it was recently shown that the bioactivity of chitosan oligomers depends on their monomer sequence [21, 23, 44, 59]. The reason for this PA dependency of biological activities of chitosans presumably lies in the presence of chitosan-specific receptors involved in the cellular perception of chitosans e.g. by the immune system of animals and plants [60].

## Three areas ripe for development

### Biotechnological production of chitosans in biorefineries and cell factories

Waste streams of the fisheries and food production are limited resources that cannot be expected to satisfy the increasing demand for well-defined chitosans [12]. There are a plethora of markets, with different requirements concerning quality and purity, different price sensitivities, and different needs in terms of volumes. While only rather small amounts (perhaps in the range of tens of kg/a) will be required for biomedical, pharmaceutical, or biocosmetic applications, the amounts required for agricultural purposes or food and feed applications are much higher (in the range of hundreds of t/a), and the demands for material applications such as for paper and textile finishing, bioplastics, or green batteries are completely beyond reach even if fungal and insect waste streams would be exploited (in the range of thousands of t/a, a single paper factory may require roughly the current global annual chitosan production of perhaps 5,000 t/a). Consequently, biotechnological production processes will be needed, also to protect biological sources from over-exploitation.

Chitosan polymers may best be produced by fungal fermentation given that fungi are natural producers of chitosans, but fungal chitosan is covalently embedded into the cell wall, making extraction difficult [15]. Genetic engineering may be employed to reduce or eliminate chitin-glucan crosslinks and the mutants might still be viable under the controlled conditions of a fermenter but so far, this approach has not yet been successful [61]. Alternatively, organisms not naturally producing chitin or chitosan may be developed into transgenic production systems secreting the polymers into the medium. Again, this has not yet been achieved at any reasonable scale [62]. Still, giving our increasing knowledge on chitin synthases and chitin deacetylases, both approaches would seem like challenges that can be met within the next decade.

Biotechnological production of defined chitosan oligomers might be more easily achieved, using microbial cell factories. Proof of principle for this approach was already shown decades ago with the recombinant expression of a bacterial chitin oligomer synthase yielding chitin pentamer in *E. coli* [63]. Co-expression with a bacterial chitin deacetylase yielded the mono-deacetylated chitosan pentamer with the GlcN unit exclusively located at the non-reducing end [64]. Varying or engineering the enzymes expressed might yield chitin and chitosan oligomers with different DP, *F*_A_, and PA. Eventually, this approach could give access to monoclonal chitosan oligosaccharides, and the process would be rather easily scalable [65].

### Interactions of bioactive chitosans with receptors and targets to understand their modes of action

Two different hypotheses have been put forward to explain the modes of action of bioactive chitosans, the ‘receptor hypothesis’ and the ‘target hypothesis’. Chitosan oligomers are likely to be recognised by specific receptors, inducing specific cellular responses, whereas polymers may rather interact non-specifically with target structures, such as polyanionic cell surfaces, unless they are quickly degraded to oligomers [44, 58, 66]. The antimicrobial activities of chitosan polymers appear to rely on target interactions with e.g. teichoic acids of Gram-positive bacteria or phospholipid membranes of fungal cells [67, 68]. However, it is unclear why such an effect seems to be lacking in other eukaryotic cells. In both plant and human cells, chitin oligomer receptors or binding proteins have been described, such as CERK1/LYK4/LYK5/CEBiP in Arabidopsis and rice plants or TLR2 in human cells, but a chitosan oligomer receptor is still unknown, though likely to exist [44, 45, 69–71]. The availability of structurally well-defined chitosan oligomers and polymers with non-random PA will enable studies into the molecular and cellular modes of action of chitosans in these different biological systems. And this fundamental knowledge will eventually lead to more specifically targeted and more reliably effective biotechnological applications of chitosans in many fields, including biomedical and pharmaceutical applications, human food and animal feed applications, biopesticide and biostimulant agricultural applications, etc.

### Biosynthesis, assembly, biodegradation, and structure–function relationships of chitin/chitosan-containing biomaterials

While chitin-containing biomaterials are wide-spread in nature, e.g. in insect cuticles and fungal cell walls, chitosan-containing biomaterials so far are known only from fungi [72–74]. Chitin-containing matrices cover the entire body of insects, fulfilling a broad diversity of roles, from flexible joints and hard cuticles to translucent lenses. How these different matrices are synthesised and assembled is far from being understood. And even less is known about the much rarer chitosan-containing fungal cell walls in which chitosan typically is a very minor component only, with unknown functions. In some pathogenic fungi, conversion of chitin to chitosan has been hypothesised to serve as a sort of stealth strategy to hide from the chitin-triggered immune system of the plant and animal hosts [18]. The only fungi thought to contain substantial amounts of chitosan in their cell walls are zygomycetes fungi, but structural details of this chitosan and how it is embedded in the complex cell wall matrix are still scarce [75]. Chitin polymers are synthesised by transmembrane chitin synthases, and the nascent chitin chains emanating from the synthases may be partially deacetylated by chitin deacetylases acting in concert with the synthases [20]. Extensive deacetylation would yield chitosan polymers, but even chitin polymers are thought to be slightly deacetylated [39, 76]. Little is known about the cooperation and regulation of these processes, and even less is known on the assembly and, sometimes, biomineralization of the complex chitin-containing matrices. Clearly, chitin-binding structural proteins will be involved in this process, and it is tempting to speculate that local deacetylations may play a crucial role in the chitin-protein interaction, given that a deacetylated GlcN unit or perhaps a small partially deacetylated domain with a specific sequence of GlcN and GlcNAc units within an otherwise fully acetylated chitin chain would represent an unmistakable marker for specific interactions. At this stage, this is fully hypothetical, but the tools and materials are rapidly becoming available to critically test such a hypothesis.

## Conclusions

**Fig. 1:**
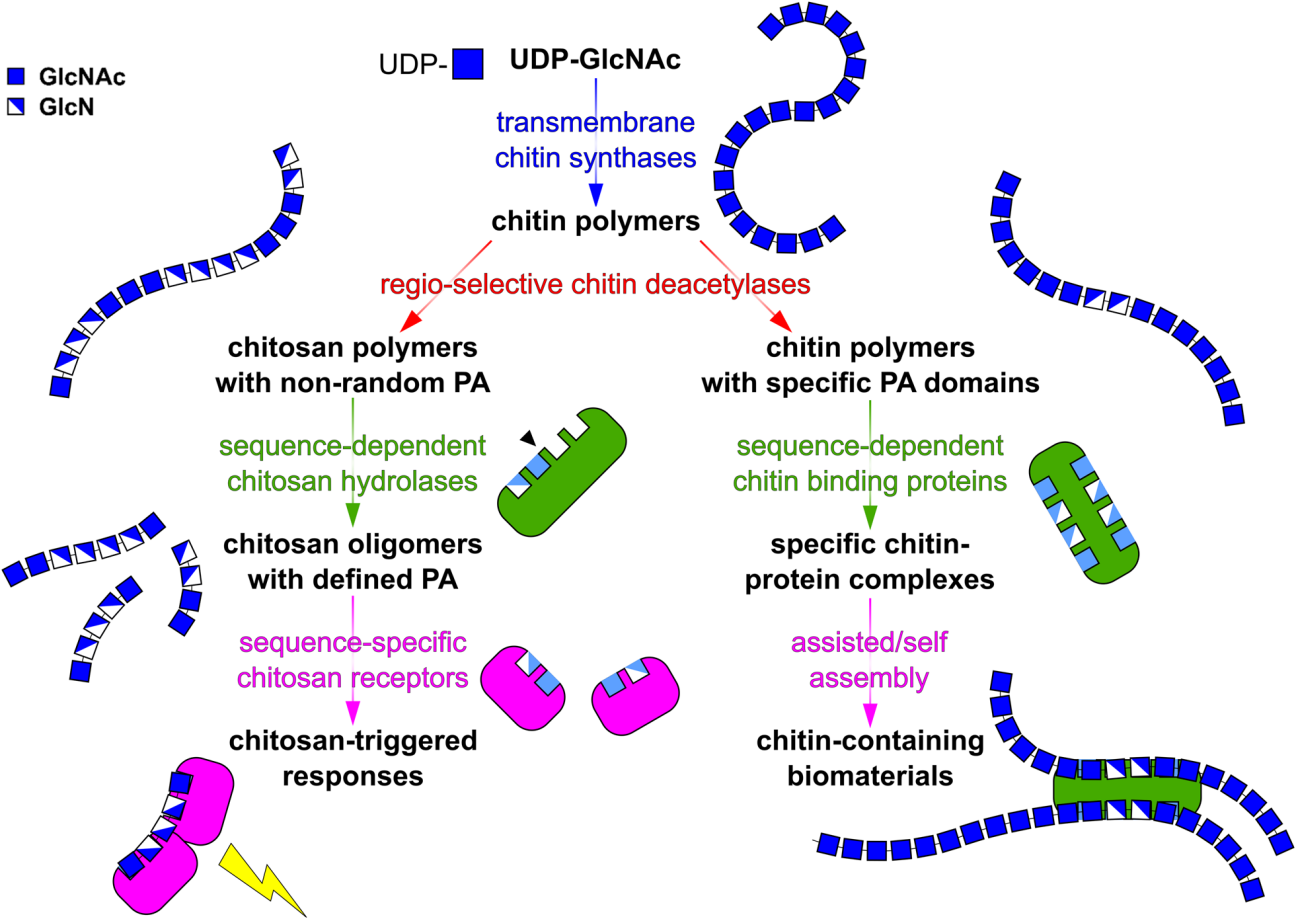
The Chito-Code: fiction today, fact tomorrow? While chitosan-specific receptors for the perception of chitosan oligomers with defined patterns of acetylation (PA) acting as functional biologics triggering specific responses in target cells have not yet been described, and while the involvement of partially deacetylated domains with specific PAs within otherwise fully acetylated chitins in the protein-assisted assembly of functional biomaterials is fully hypothetical, regio-selective chitin deacetylases and sequence-dependent chitosan hydrolases and chitin-binding proteins are well known

Chitins and chitosans and the matrices of which they are integral components are so much more than simple structural protections for delicate cells and organisms. They are functional biomaterials as well as bioactive biologics. In both respects, their versatility is expressed by specific interactions with proteins which are responsible for their biosynthesis, modification, assembly, perception, and degradation. The PA of chitosans may act like a ‘chito-code’, ‘written’ by trans-membrane chitin synthases in conjunction with regio-selective chitin deacetylases, ‘read’ by sequence-dependent chitin binding proteins and chitosan hydrolases and, possibly, ‘understood’ by a proteinaceous assembly machinery and pattern recognition receptors (Fig. 1). Today, this is just a hypothesis, but one that can and will be tested in the foreseeable future.

## Data Availability

N/a.
